# Identification of a Novel Cuproptosis-Related Gene Signature and Integrative Analyses in Thyroid Cancer

**DOI:** 10.3390/jcm12052014

**Published:** 2023-03-03

**Authors:** Jiapeng Huang, Jinyuan Shi, Pu Wu, Wei Sun, Dalin Zhang, Zhihong Wang, Xiaoyu Ji, Chengzhou Lv, Ting Zhang, Ping Zhang, Hao Zhang

**Affiliations:** Department of Thyroid Surgery, The First Hospital of China Medical University, Shenyang 110000, China

**Keywords:** cuproptosis, thyroid cancer, prognosis, signature, TCGA

## Abstract

Cuproptosis is a novel programmed cell death that depends on copper. The role and potential mechanism of cuproptosis-related genes (CRGs) in thyroid cancer (THCA) are still unclear. In our study, we randomly divided THCA patients from the TCGA database into a training set and a testing set. A cuproptosis-related signature consisting of six genes (SLC31A1, LIAS, DLD, MTF1, CDKN2A, and GCSH) was constructed using the training set to predict the prognosis of THCA and was verified with the testing set. All patients were classified into low- and high-risk groups according to risk score. Patients in the high-risk group had a poorer overall survival (OS) than those in the low-risk group. The area under the curve (AUC) values for 5 years, 8 years, and 10 years were 0.845, 0.885, and 0.898, respectively. The tumor immune cell infiltration and immune status were significantly higher in the low-risk group, which indicated a better response to immune checkpoint inhibitors (ICIs). The expression of six cuproptosis-related genes in our prognostic signature were verified by qRT-PCR in our THCA tissues, and the results were consistent with TCGA database. In summary, our cuproptosis-related risk signature has a good predictive ability regarding the prognosis of THCA patients. Targeting cuproptosis may be a better alternative for THCA patients.

## 1. Introduction

Thyroid carcinoma (THCA) is the most common malignancy of the endocrine system, and its global incidence has been increasing dramatically in the last 30 years [1]. The incidence of THCA ranked ninth in the global cancer statistics in 2020. As the third most common female malignant tumor in China, the overall incidence rate of THCA in females is three times greater than that in males [1,2]. Papillary thyroid carcinoma (PTC) is the most frequent pathological subtype of THCA, which was the main contributor to the rapidly increasing rate and has consistently increased in all countries [3]. After standard surgery, TSH suppression, and radioiodine therapy, most PTC patients have a relatively ideal prognosis. However, there are still some patients with recurrence and distant metastasis after surgery, which seriously affects their quality of life and survival rate [4,5]. Therefore, further studies are required to clarify the mechanism of the occurrence and development of thyroid cancer and screen novel prognostic genes or construct promising prognostic models.

Copper (Cu) is an indispensable metallic element for life. As a redox-active cofactor, Cu plays a significant role in maintaining the various biological functions of organisms in the human body, such as aerobic respiration, energy production, signal transduction, etc. [6,7]. Similarly, to other trace metals necessary for the human body, an imbalance of Cu homeostasis in cells may cause irreversible damage to cells and be involved with Menkes disease, Alzheimer’s disease, and cardiovascular disease [8,9]. It could cause cell death through the accumulation of reactive oxygen species, proteasome inhibition, anti-angiogenesis, and other mechanisms [10]. The latest research has found that excessive Cu can regulate cell death through an unknown mechanism that is different from apoptosis, ferroptosis, and necroptosis, recently termed “cuproptosis”. They reported that copper-dependent cell death mainly occurred through directly binding to lipoylated mitochondrial enzymes of the tricarboxylic acid (TCA) cycle, which leads to proteotoxic stress and, ultimately, cell death [11]. 

With the further study of copper uptake and transport-related proteins, experts have found that Cu plays an important role in mitochondria respiration, immune regulation, and antioxidant defense against cancers. Recently, it has been found that copper is the key factor for the rapid growth of cancer cells and participates in various oncogenic signaling. Therefore, targeting the metabolic vulnerabilities of copper is becoming a novel anti-cancer treatment strategy [12,13]. Elesclomol, which was previously used as an anticancer drug that targets mitochondrial metabolism, was later found to inhibit tumors by causing cuproptosis [14]. It is reported that Disulfiram (DSF) can combine with copper to target cancer stem cells (CSCs) in various cancers. Recently, Ni et al. [15] found that DSF/copper can inhibit the proliferation of DTC cell lines by suppressing thyrosphere formation, indicating that targeting copper metabolism may be a new therapeutic strategy for DTC. In addition, the level of Cu in urine is negatively correlated with the risk of THCA [16]. 

In this study, we focused on whether these cuproptosis-related genes (CRGs) could predict the prognosis of THCA patients. In our study, we aimed to construct a prognostic signature of CRGs for THCA. We propose the cuproptosis-related signature in THCA for the first time, aiming to provide a sufficient basis for further mechanism research in the future. We further explored the association between the prognostic signature and tumor-infiltrating immune cells of patients with THCA, which may help to predict the response of patients with different risk categories to immunotherapy individually.

## 2. Materials and Methods

### 2.1. Data Collection and Clinical Samples

We downloaded the gene expression profiles and corresponding clinical information of 507 patients with THCA from the TCGA database (https://portal.gdc.cancer.gov/, accessed on 18 May 2022). The data included 58 non-diseased samples and 510 papillary thyroid cancer samples. The patients with incomplete data were excluded; then, the remaining 502 patients (the entire set) were randomly divided into a training set (*n* = 251) and a testing set (*n* = 251). The clinical characteristics of all patients with THCA are shown in Table 1, and there was no statistical difference in clinical data between the two groups.

### 2.2. Differentially Expressed Genes (DEGs) Identification

In total, 15 CRGs, including GLS, CDKN2A, ATP7B, ATP7A, MTF1, SLC31A1, DLD, DLAT, PDHA1, PDHB, FDX1, GCSH, LIAS, LIPT1, and LIPT2, were obtained from the reviews [11]. We identified DEGs in all tumor and normal samples using the “limma” package in R software. A *p*-value < 0.05 and |log2 fold change|> 1) were set as the screening criteria. The DEGs were signed with * if *p* < 0.05, ** if *p* < 0.01, and *** if *p* < 0.001.

### 2.3. Construction of the Protein–Protein Interaction (PPI) Network

A PPI network was constructed using the Search Tool for the Retrieval of Interacting Genes/Proteins (STRING) database (https://cn.string-db.org/, accessed on 8 June 2022) to investigate the interactions between these DEGs. 

### 2.4. Univariate Cox Regression Analysis

A univariate Cox proportional hazards regression was used to identify the prognostic values of CRGs. To prevent omission, a *p*-value < 0.2 was set as the cut-off to determine prognostic variables. 

### 2.5. Consensus Clustering of Prognostic CRGs Genes

According to the prognostic CRGs, patients with THCA were clustered into distinct subgroups using the “ConsensusClusterPlus” package in R language. The survival probability of different clusters was analyzed using the Kaplan–Meier curves of overall survival (OS). 

### 2.6. Construction of Cuproptosis-Related Prognostic Signature

Data from the training set were used to construct the cuproptosis-related prognostic model, while the testing set and the entire set were used to validate its predictive capability. Firstly, we used a least absolute shrinkage and selection operator (LASSO) Cox regression analysis to minimize overfitting and constructed the cuproptosis-related prognostic signature with the “glmnet” package [17]. The model was ascertained by the penalty parameter (λ), which corresponded to the partial likelihood deviance and was tested with tenfold cross-validation. The risk score of each patient was determined by the gene expression level and its coefficient. The risk score was calculated as follows: risk score = sum (each CRG expression level × its corresponding coefficient). Subsequently, all the patients were classified into low- and high-risk groups according to the median risk score. Finally, we used Kaplan–Meier survival curves, receiver operating characteristic (ROC) curves, and the value of the area under the curve (AUC) to evaluate the predictive ability of the prognostic signature for OS in all sets. Univariate and multivariate Cox regression analyses were also used to determine independent prognostic variables for OS in all sets. 

### 2.7. The Clinicopathologic Features between the Low- and High-Risk Groups

To analyze the differences in prognoses between low- and high-risk groups with different clinical features, patients were divided into distinct subgroups according to the clinicopathologic features, including age, gender, and TNM stage. Kaplan–Meier survival analysis was performed in different subgroups through the ‘’survival’’ and ‘’survminer’’ packages. The “limma” and “heatmap” packages were used to build a heatmap clarifying the relationship between risk scores and clinicopathologic features. We also conducted subgroup analyses on patients with different ages, genders, and TNM stages to determine whether the CRGs still had the same predictive value as our model. 

### 2.8. Principal Component Analysis (PCA) 

In order to reflect the distinction between the low- and high-risk groups, we used the “stats” package to perform PCA for CRGs and risk genes. 

### 2.9. Functional Enrichment Analysis

According to the prognostic signatures, all samples were classified into low- and high-risk groups. Gene Ontology (GO) and Kyoto Encyclopedia of Genes and Genomes (KEGG) enrichment analyses were performed with the “clusterProfiler” package according to the DEGs (|log2FC| ≥ 1 and FDR < 0.05). Meanwhile, gene set enrichment analysis (GSEA) was performed in the Hallmark gene set “h.all.v2022.1.Hs.symbols.gmt” to analyze the enriched biological pathways of key genes using GSEA 4.2.2. 

### 2.10. Immune-Related Analysis

The immune score, stromal score, ESTIMATES score, and tumor purity in each tumor sample were estimated by the ESTIMATE algorithm using the “estimate” package [18]. Then, the differences in the scores were compared between the low- and high-risk groups. Moreover, we performed single-sample GSEA (ssGSEA) to analyze the correlation between the expression level of prognostic CRGs and the immune infiltration degree of 23 immune cell types and calculate the scores of 13 immune-related pathways using the “GSVA” package in the low- and high-risk groups [19]. The expression of the HLA genes was also compared between the two groups. In addition, we calculated the abundance of 23 types of infiltrating immune cells in different cluster groups according to THCA transcriptional profiles. 

### 2.11. Immunophenoscore (IPS) Analysis

Major histocompatibility complex (MHC) molecules, effector cells, suppressive cells, and immunomodulators determined the immunogenicity. Each patient’s IPS was calculated based on the expression of the representative genes by machine learning [20]. The IPS values of each THCA patient were downloaded from The Cancer Immunome Atlas (https://tcia.at/home, accessed on 18 December 2022) and positively correlated with tumor immunogenicity. The IPS values were then used to compare the response to immune checkpoint inhibitors (ICIs) between the low- and high-risk groups. 

### 2.12. Potential Targeted Drug Sensitivity Prediction 

In order to screen potential therapeutic drugs, we used the “pRRophetic” R package to predict the sensitivity toward targeted therapy from the gene expression and drug sensitivity data. 

### 2.13. Total RNA Extraction and qRT-PCR

In total, 50 paired PTC tissue samples and their adjacent non-cancerous thyroid tissue samples were obtained from the Thyroid Surgery Department of the First Hospital of China Medical University. Total RNA was extracted from frozen specimens obtained from tissue samples and cells using RNAiso Plus (Takara, Kusatsu City, Japan). Reverse transcription was performed using PrimeScript™ RT Master Mix; qRT-PCR was performed using SYBR Premix Ex Taq II (Takara, Japan) on a Light Cycle0r 480 system (Roche, Nutley, NJ, USA). Primer sequences used in the current study are shown in Table S1. The 2^−ΔΔCt^ method was used to calculate the relative expression levels (CT, cycle threshold). GAPDH was used as an internal control. 

### 2.14. Statistical Analysis

All the data were analyzed using Bioconductor packages in R software (version 4.1.3). The difference in the expression of CRGs between non-diseased samples and tumor samples was compared using the Wilcoxon rank-sum test. Univariate and multivariate Cox regression analyses were performed to predict prognosis in THCA patients. The *p*-value < 0.05 was set as statistically significant for all the analyses.

## 3. Results

### 3.1. Identification of DEGs in THCA from the TCGA Database

**Figure 1:**
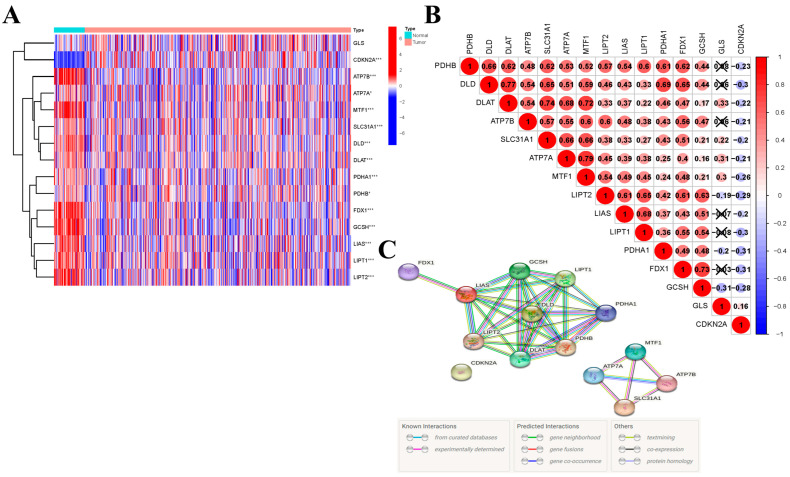
Expression of the cuproptosis-related genes in thyroid carcinoma (THCA). (**A**) The heatmap showed the expression levels of 15 cuproptosis-related genes in normal and tumor samples. * if *p* < 0.05, and *** if *p* < 0.001. (**B**) Pearson correlation analysis of the 15 cuproptosis-related genes in THCA. (**C**) PPI network indicated the interactions of the cuproptosis-related genes.

According to the *p*-value < 0.05, 14 differentially expressed genes were identified from 15 CRGs. Among them, 13 were down-regulated (ATP7B, ATP7A, MTF1, SLC31A1, DLD, DLAT, PDHA1, PDHB, FDX1, GCSH, LIAS, LIPT1, and LIPT2) and only CDKN2A was up-regulated in THCA tumors. The RNA expression levels of these genes are shown in the heatmap (Figure 1A). Subsequently, we analyzed the relationship between these genes; a significant correlation existed between most genes. We found that ATP7A was significantly positively correlated with MTF1 (Cor = 0.79), while CDKN2A and PDHA1, and FDX1 were significantly negatively correlated (Cor = −0.31) (Figure 1B). A PPI network was constructed to illustrate the interactions among these differentially expressed CRGs (Figure 1C).

### 3.2. Consensus Clustering of Cuproptosis-Related Genes

**Figure 2:**
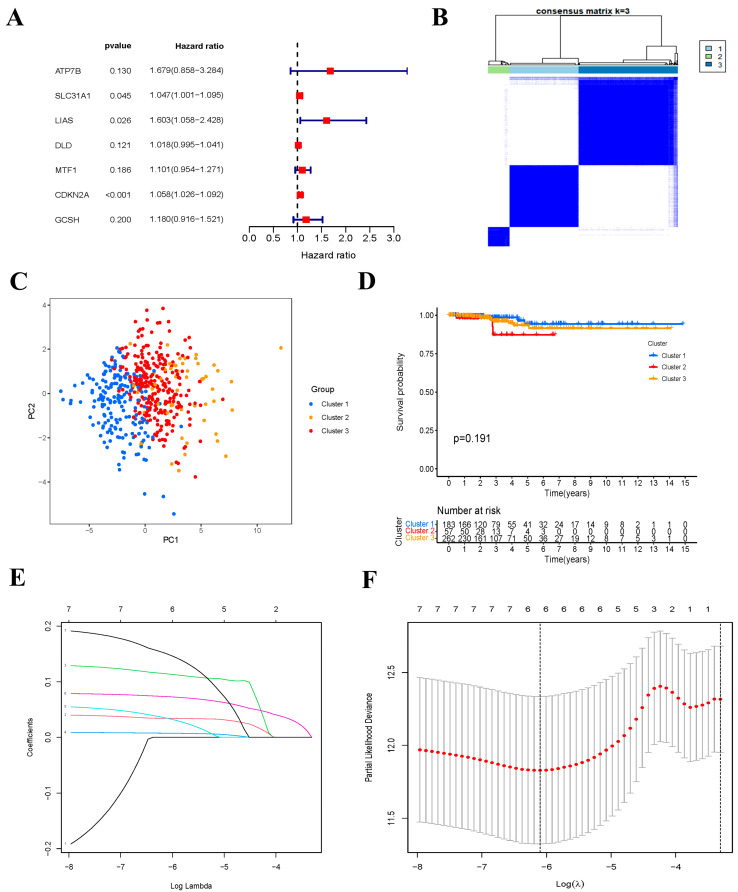
Univariate Cox regression analysis, identification of consensus clusters based on the cuproptosis-related genes and LASSO analysis. (**A**) Forest plot showing the result of univariate Cox regression analysis of OS, seven genes with *p* < 0.2. (**B**) Consensus clustering matrix for k = 3. (**C**) Principal Component Analysis (PCA) of the RNA expression profile in TCGA cohort. (**D**) Kaplan–Meier curves of overall survival (OS) in three clusters. (**E**) Cross-validation for tuning parameter selection in the LASSO regression. (**F**) LASSO analysis of seven prognostic cuproptosis-related genes.

First, we performed a univariate Cox regression analysis to identify the prognostic CRGs. Seven genes meeting the criterion of a *p*-value < 0.2 were significantly correlated with OS. All the genes (ATP7B, SLC31A1, LIAS, DLD, MTF1, CDKN2A, and GCSH) were risk genes with HR > 1 (Figure 2A). To investigate the relationships between the expression of the cuproptosis-related DEGs and THCA subtypes, we performed a consensus clustering analysis based on the seven cuproptosis-related DEGs. We found that an amount of three clusters was the optimal clustering stability from two to nine. Subsequently, the THCA patients were clustered into three subtypes: Cluster 1 (*n* = 183), Cluster 2 (*n* = 57), and Cluster 3 (*n* = 262) (Figure 2B). We also analyzed the gene expression pattern between three subtypes of PCA. The results suggested that Cluster 1, Cluster 2, and Cluster 3 could gather together (Figure 2C). There were no statistical differences between the three clusters and OS (Figure 2D).

### 3.3. Construction of Cuproptosis-Related Risk Signature

**Figure 3:**
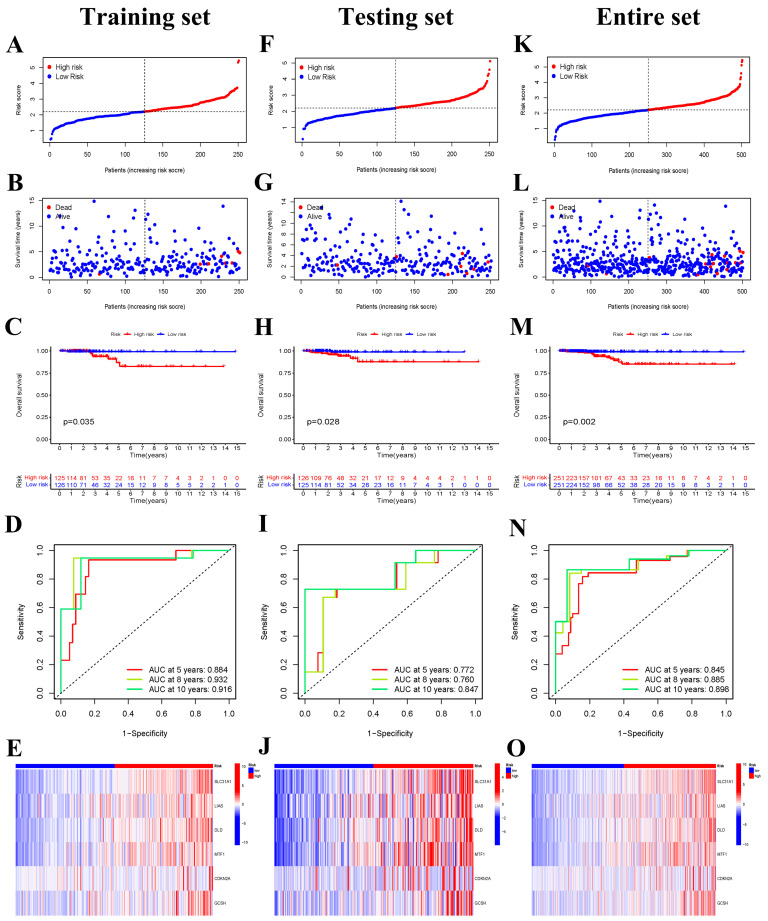
Identification of six cuproptosis-related genes prognostic signatures in the training set, the testing set, and the entire set. (**A**) The distribution of risk scores, (**B**) survival status, (**C**) Kaplan–Meier curve analysis of overall survival of THCA in low− and high−risk groups, (**D**) time-dependent ROC analysis, and (**E**) the expression of six prognostic CRGs in low- and high-risk groups in the training set. (**F**) The distribution of risk scores, (**G**) survival status, (**H**) Kaplan–Meier curve analysis of overall survival of THCA in low− and high−risk groups, (**I**) time−dependent ROC analysis, and (**J**) six-CRGs expression patterns in low- and high-risk groups in the testing set. (**K**) The distribution of risk scores, (**L**) survival status, (**M**) Kaplan–Meier curve analysis of overall survival of THCA in low− and high−risk groups, (**N**) time−dependent ROC analysis, and (**O**) six-CRGs expression patterns in low− and high−risk groups in the entire set.

To minimize overfitting, LASSO Cox regression analysis was conducted based on the seven differentially expressed prognostic CRGs, and a six-gene risk signature was constructed according to the optimum λ value (Figure 2E,F). The formula of the six-gene signature was as follows: risk score = (0.0342 × SLC31A1) + (0.1142 × LIAS) + (0.0075 × DLD) + (0.0343 × MTF1) + (0.0732 × CDKN2A) + (0.1499 × GCSH). The risk scores of all patients in the training set were calculated according to the above formula, and then the median of these risk scores was also determined. Subsequently, patients were classified as the low-risk group if their risk score was lower than the median risk score, otherwise patients were classified as the high-risk group. Firstly, we ranked the patients’ risk scores and analyzed their distributions in the training set (Figure 3A). The survival status of THCA patients in the training set was shown in the scatter diagram (Figure 3B). Kaplan–Meier survival curves showed that patients in the high-risk group had a significantly poorer OS than patients in the low-risk group (*p* = 0.035) (Figure 3C). Time-dependent ROC curves confirmed the predictive effect of the signature-based risk score for OS. The areas under the curve (AUC) values of the risk signature were 0.884 for the 5-years OS, 0.932 for the 8-years OS, and 0.916 for the 10-years OS, respectively (Figure 3D). In addition, the expression patterns of six prognostic genes in two risk groups is shown in the heatmap (Figure 3E). 

Furthermore, the predictive performance of the risk signature was further verified in the testing set and the entire set. The risk scores of each patient were calculated and the patients were classified into low- and high-risk groups in the two sets based on our risk signature. The distribution of the risk score and survival status in the testing set were shown in Figure 3F,G. Kaplan–Meier survival curves also showed that the OS of high-risk patients was lower than that of the low-risk group in the testing set (*p* = 0.028) (Figure 3H). The AUCs were 0.772, 0.760, and 0.847 for 5 years OS, 8 years OS, and 10 years OS, respectively (Figure 3I). The expression patterns of six CRGs were shown in Figure 3J. The results in the entire set were consistent with those in the training set and testing set. The distribution of risk scores and patients’ survival status are also presented in Figure 3K,L. The OS in the high-risk group was also significantly lower than that in the low-risk groups (*p* = 0.002) (Figure 3M). The AUC values were 0.845, 0.885, and 0.898 for 5 years OS, 8 years OS, and 10 years, respectively (Figure 3N). The expression heatmap of six prognostic genes is displayed in Figure 3O. 

### 3.4. The Six-Gene Risk Signature Is an Independent Prognostic Indicator for OS

**Figure 4:**
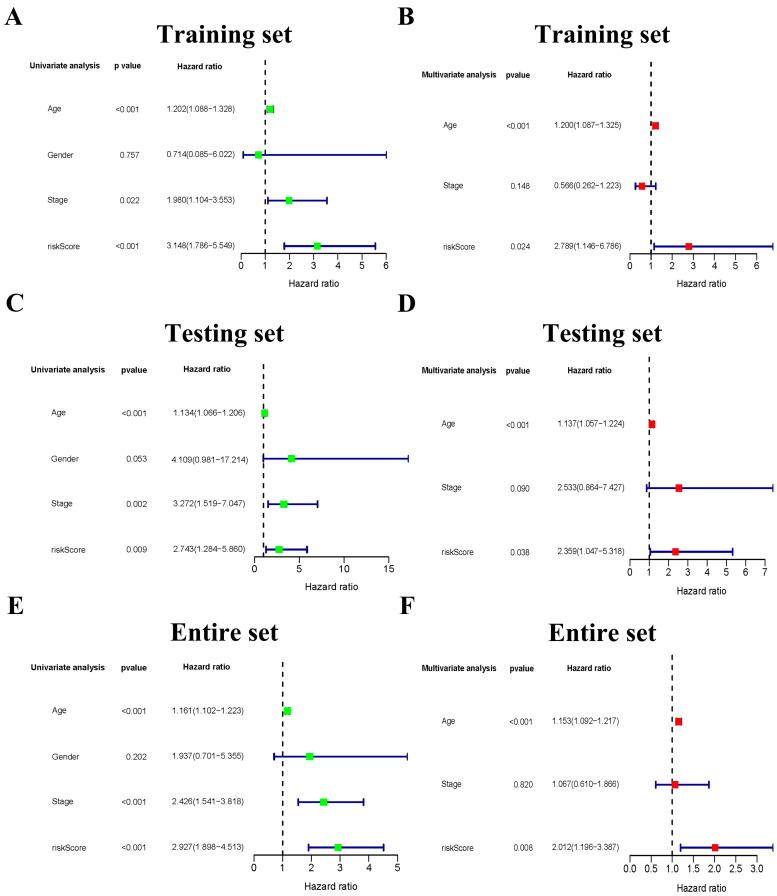
The association between clinicopathological factors and OS in patients with THCA. Univariate Cox regression analyses (**A**) and multivariate Cox regression analyses (**B**) of the association between clinicopathological factors and OS in the training set. Univariate Cox regression analyses (**C**) and multivariate Cox regression analyses (**D**) of the association between clinicopathological factors and OS in the testing set. Univariate Cox regression analyses (**E**) and multivariate Cox regression analyses (**F**) of the association between clinicopathological factors and OS in the entire set.

Univariate and multivariate Cox regression analyses were performed to investigate the available variables on OS of THCA patients and confirmed that the risk score was an independent prognostic predictor for OS in the training set (Figure 4A,B; univariate: HR = 3.148, 95% CI = 1.786–5.549, *p* < 0.001; multivariate: HR = 2.789, 95% CI = 1.146–6.786, *p* = 0.024). These two analyses were also carried out in the testing set and the entire set, obtaining the same conclusion as the training set. The HR values of the univariate analysis and multivariate analysis were 2.743 (CI =1.284–5.860, *p* = 0.009) and 2.359 (CI =1.047–5.318, *p* = 0.038) in the testing set (Figure 4C,D). Similarly, The HR values of these analyses were 2.927 (CI =1.898–4.513, *p* < 0.001) and 2.012 (CI =1.196–3.387, *p* = 0.008) in the entire set (Figure 4E,F).

### 3.5. The Clinicopathological Stratified Analysis Validates the Risk Signature’s Prediction Performance 

We analyzed the difference in risk scores between subgroups with different clinicopathological characteristics. The results showed that there were significant differences between the different ages and N stages. The risk score was significantly higher in patients aged ≥55 and N0 stage (Figure S1A,D). However, the risk score had no statistical difference between the different gender, the T stage, the M stage, and the clinical stage in our risk signature (Figure S1B,C,E,F). The heatmap indicated the significant differences in the N stage and cluster between the low- and high-risk group (Figure S1G). In addition, clinicopathological stratified analysis was performed to validate the prediction performance of our risk signature. There was no significant difference between the low- and high-risk groups in the OS of four subgroups (patients aged <55, N1, M0, and M1), while the other eight subgroup analyses (age ≥ 55, female, male, T1-2, T3-4, N0, stage I–II, and stage III–IV) indicated that the patients in the high-risk group had a poorer prognosis compared with those in the low-risk group (Figure S2).

### 3.6. Functional Analyses Based on the Risk Signature

**Figure 5:**
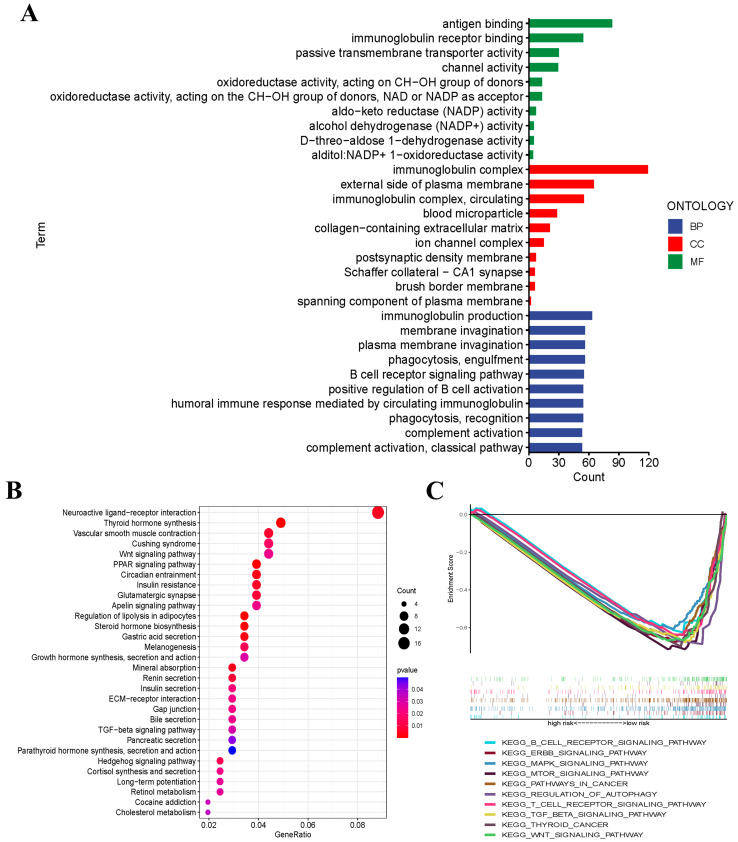
Functional enrichment analysis based on the DEGs. (**A**) Barplot graph for GO enrichment. (**B**) Bubble graph for KEGG pathways. (**C**) Gene set enrichment analysis (GSEA) showed the significantly enriched hallmarks of tumor sets based on the risk signature in TCGA.

To further clarify the potential biological functions and signaling pathways that are related to the risk score, GO enrichment and KEGG pathway analyses were conducted according to the six genes of risk signature between the low- and high-risk groups. The results suggested that the DEGs were mainly enriched in immune response, neuroactive ligand–receptor interaction, and thyroid hormone synthesis (Figure 5A,B).

### 3.7. Gene Set Enrichment Analysis (GSEA)

GSEA was performed to examine the transcript messages of THCA patients who were grouped by risk score into low- and high-risk groups. The results demonstrated that the cuproptosis-related prognostic signature genes regulated multiple signaling pathways of thyroid cancer. In detail, biological pathways such as the B-cell receptor, ERBB, MAPK, and MTOR signaling pathways were enriched in the low-risk group. (Figure 5C). 

### 3.8. Immune Function and Immune Status Analyses between the Low- and High-Risk Groups

**Figure 6:**
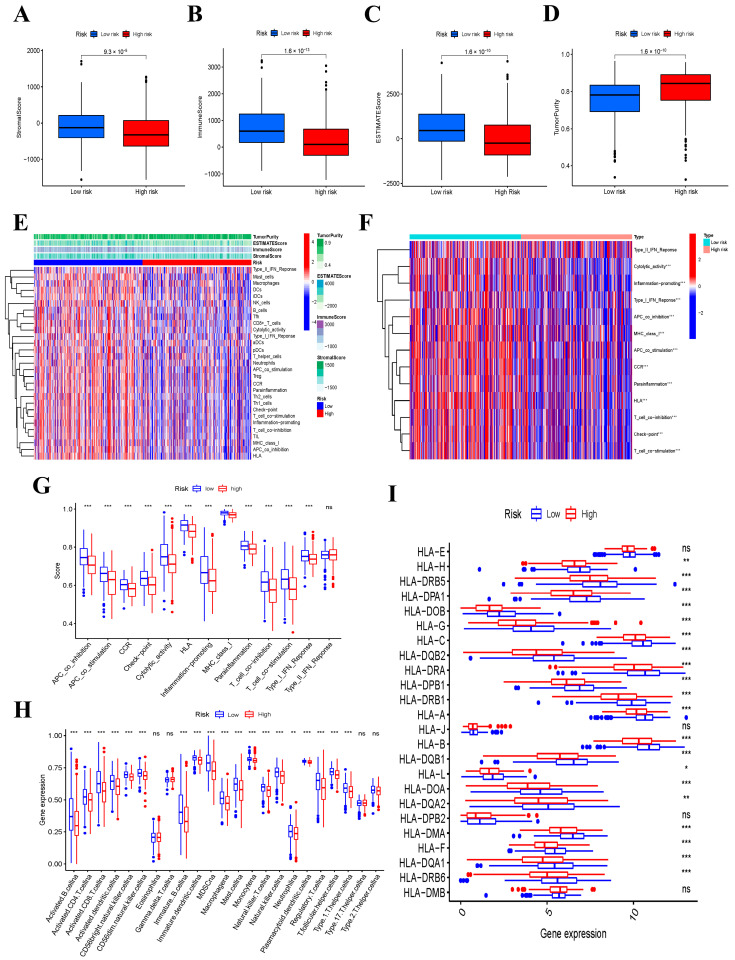
Comparison of the ssGSEA scores in the low− and high−risk groups. * *p* < 0.05; ** *p* < 0.01; *** *p* < 0.001; and ns not significant. (**A**) Stromal scores, (**B**) immune stores, (**C**) ESTIMATE scores, (**D**) tumor purity between low− and high−risk groups. (**E**) The immune status between low− and high−risk groups, (**F**,**G**) 13 immune−related functions, (**H**) abundance of 23 infiltrating immune cell types, and (**I**) the expression of HLA−related genes between high− and low−risk groups.

Each patient’s stromal score, immune score, and ESTIMATE score were calculated to reflect the number of stromal and immune cells in their tumor sample. The results showed that the low-risk group contained more stromal cells and immune cells than the high-risk group, and the ESTIMATE score was correspondingly greater in the low-risk group (Figure 6A–C). In contrast to the immune activities, the tumor purity was lower in the low-risk group (Figure 6D). In order to evaluate the relationship between the immune status and the risk score, we calculated the infiltrating scores of 16 immune cells and the activity of 13 immune-related pathways between the low- and high-risk groups in THCA patients of the TCGA cohort using the ssGSEA. The heatmap indicated the immune status of 29 immune signature gene sets in the two groups (Figure 6E). In addition, the heatmap and boxplot showed that the immune-related pathways had significantly higher activity in the low-risk group than those in the high-risk group (Figure 6F,G). Subsequently, we evaluated the relationship between the two groups and the 23 subtypes of immune cells to further explore how these six genes affect the tumor microenvironment. According to our results, there is a significant difference between the two groups regarding the amount of infiltration in most immune cells (Figure 6H). Finally, we analyzed the expression levels of HLA-related genes; the results showed that the expression levels of most HLA genes in the low-risk group were higher than those in the high-risk group (Figure 6I). 

### 3.9. IPS Analysis and Response to ICIs

**Figure 7:**
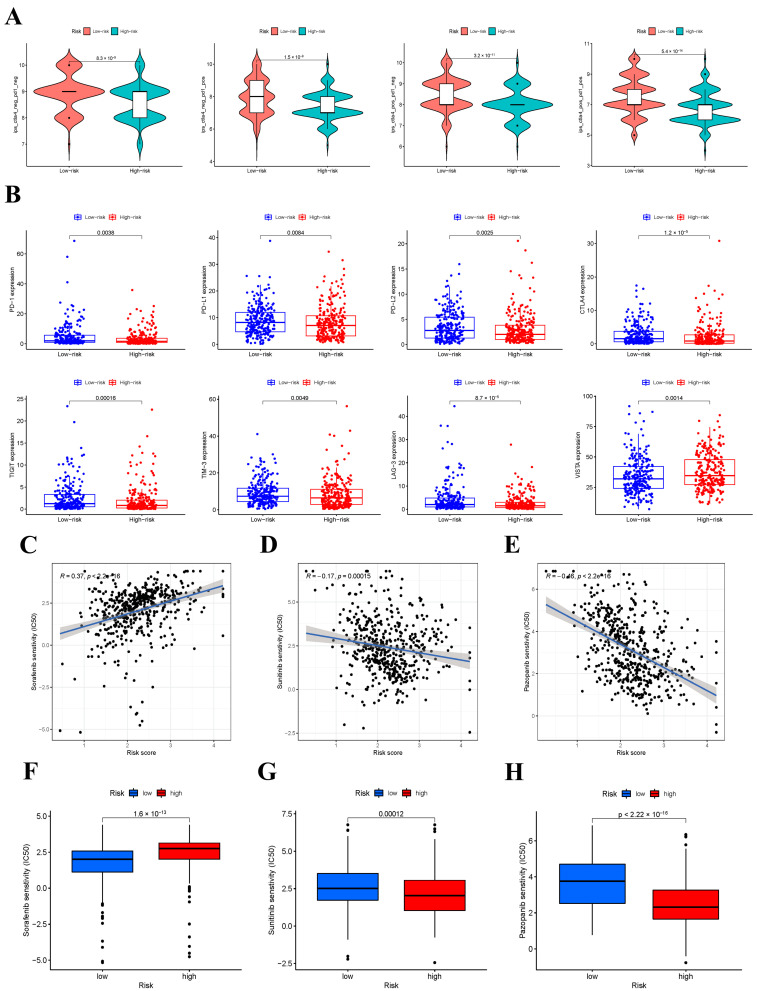
IPS analysis and the role of the prognostic risk score in targeted therapy. (**A**) The association between IPS and the immune−related prognostic signature. (**B**) The expression of PD−1, PD−L1, PD−L2, CTLA4, TIGIT, TIM−3, LAG−3, and VISTA in low− and high−risk groups. (**C**–**E**) The correlation between risk scores and estimated IC50 value of sorafenib (**C**), sunitinib (**D**), and pazopanib (**E**). (**F**–**H**) Comparison of estimated IC50 value of sorafenib (**F**), sunitinib (**G**), and pazopanib (**H**) between low− and high−risk score groups.

A crucial role of IPS is to predict the response to ICIs in THCA patients; therefore, we explored the relationship between IPS and our risk signature. IPS, IPS-PD-1/PD-L1/PD-L2, IPS-CTLA4, and IPS-PD-1/PD-L1/PD-L2 + CTLA4 scores were used to estimate the response to ICIs in THCA patients. The IPS, IPS-PD-1/PD-L1/PD-L2, IPS-CTLA4, and IPS-PD-1/PD-L1/PD-L2 + CTLA4 scores were significantly higher in the low-risk group for OS (Figure 7A). The results demonstrated that patients in the low-risk group seemed to be more sensitive to ICI treatment. Furthermore, the expression levels of PD-1, PD-L1, PD-L2, CTLA4, TIGIT, TIM-3, and LAG-3 were higher in the low-risk group (*p* < 0.001), whereas VISTA was lower in the low-risk group (Figure 7B). Thus, the patients in the low-risk group indicated a better response to ICIs.

### 3.10. Response to Targeted Drugs 

Targeted drug therapy is also a treatment method for iodine refractory thyroid cancer and advanced thyroid [21]. The sensitivity of THCA patients in the TCGA cohort to targeted drugs was predicted by “pRRophetic”. Fortunately, we identified potentially effective targeted drugs in both low-risk and high-risk groups. We found that the half-maximal inhibitory concentration (IC50) of sorafenib was lower in the low-risk group, indicating that the patients in the low-risk group were more sensitive to sorafenib (Figure 7C,F). In contrast, the IC50 of sunitinib and pazopanib was lower in the high-risk group, illustrating that the patients in the high-risk group were more sensitive to these two drugs (Figure 7D,E,G,H). 

### 3.11. The Expression Levels of Six Prognostic Risk Genes

**Figure 8:**
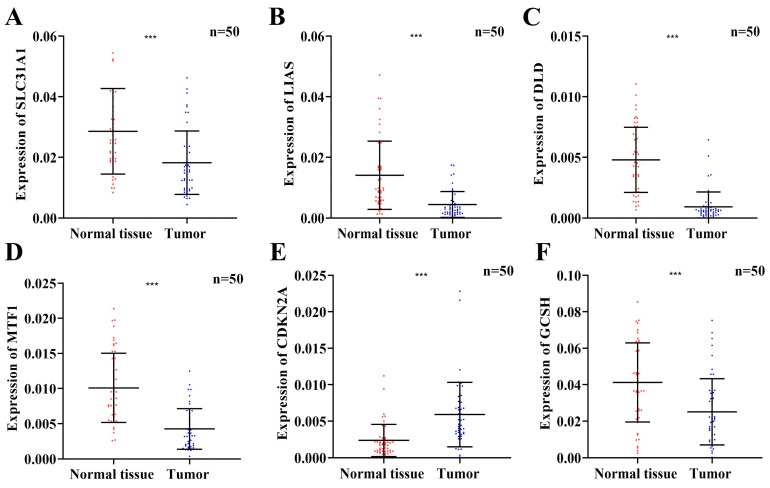
The relative expression levels of SLC31A1 (**A**), LIAS (**B**), DLD (**C**), MTF1 (**D**), CDKN2A (**E**), and GCSH (**F**) in 50 pairs of tissue samples from PTC patients by qRT-PCR. (*** *p* < 0.001).

Based on our bioinformatics analysis results, we further verified the expression of the six prognostic risk genes (SLC31A1, LIAS, DLD, MTF1, CDKN2A, and GCSH) in 50 pairs of tissue samples from PTC patients by qRT-PCR. The results showed that the mRNA expression of SLC31A1, LIAS, DLD, MTF1, and GCSH were significantly lower in PTC tissues (*p* < 0.001), while the expression of CDKN2A was significantly higher in PTC tissues (*p* < 0.001) (Figure 8). The results of the experiment were consistent with the results of our bioinformatic analysis.

## 4. Discussion

THCA is the most frequent endocrine malignancy, PTC accounts for more than 85% of them [22]. Although most PTC patients have a satisfactory prognosis, there are still some patients with recurrence, iodine refractory, distant metastasis, have other adverse outcomes after surgery, and exhibit distinct molecular features [23,24]. These patients often have a poor quality of life and inferior survival time. Therefore, it is urgent to construct a high-quality risk signature to predict the prognosis of patients, and to provide optimal treatment to patients with different risks. Simultaneously, seeking more new therapeutic targets for THCA is also a huge challenge.

Similar to iron, copper is an indispensable element for most aerobic organisms. Cuprous (Cu^1+^) and cupric (Cu^2+^) are the two main oxidation states of copper in the human body. Cu^1+^ is easily oxidized to Cu^2+^ in the presence of oxygen or other electronic receptors; thus, Cu^2+^ is the main state in the biological system [7]. As a catalytic cofactor, Cu plays a pivotal role in the redox chemistry of enzymes, mitochondrial respiration, and modulating angiogenin activity [7,25]. Cu homeostasis is strictly maintained by copper uptake, transport, and excretion in the cell and between the intercellular spaces. The disruption of Cu homeostasis can cause cell dysfunctions and then lead to the occurrence of many diseases. Once the homeostasis of Cu is disrupted, it will initiate oxidative damage, lipid peroxidation, alpha-synuclein aggregation, etc. [26]. Significantly, more and more studies have recognized that Cu plays a vital role in the development and progression of various cancers, such as cell proliferation, angiogenesis, and carcinogenesis [27,28,29]. In addition, aberrantly elevated serum copper may be related to the occurrence and recurrence of cancers [30]. At the same time, Cu is becoming the new target for targeted therapy in cancers [31,32]. Recently, a new form of cell death caused by Cu was discovered, which was termed “cuproptosis”. The occurrence of cuproptosis mainly regulates mitochondrial respiration. In detail, excessive Cu in the cells could be transported to the mitochondria through ionophores and bind directly to lipoylated components of the tricarboxylic acid (TCA) cycle, resulting in aggregation of the lipoylated protein and the loss of iron–sulfur cluster proteins, subsequently leading to proteotoxic stress and, ultimately, to cell death [11]. This breakthrough rapidly made cuproptosis a research hotspot, providing new inspiration for risk prediction and targeted treatment in cancer. More importantly, Cu is essential for oncogenic BRAF signaling and tumorigenesis; BRAF mutation is the most common genetic change in THCA [33]. Therefore, it is vital to study the role of cuproptosis in THCA.

In our study, we first analyzed the mRNA expression levels of 15 cuproptosis-related genes in normal samples and THCA samples, and all genes except GLS were differentially expressed. Among these genes, 13 were down-regulated and only CDKN2A was up-regulated in THCA samples, with all of them showing significant correlation. Then, we obtained seven prognostic risk genes from these differentially expressed CRGs through univariate Cox regression analysis. Unfortunately, when we used these seven genes for consensus cluster analysis, although we identified three completely different subgroups, there was no significant difference in the prognosis of the three subgroups. Subsequently, we constructed a prognostic risk signature consisting of six cuproptosis-related genes (SLC31A1, LIAS, DLD, MTF1, CDKN2A, and GCSH) from these prognostic CRGs through LASSO Cox regression analysis. Moreover, the prognostic value of the six prognostic risk signatures was evaluated and verified in the TCGA internal dataset. SLC31A1, also called CTR1 (copper transporter 1), is a key copper importer that mediates high-affinity cellular copper entry, which exerts an important role in maintaining intracellular copper homeostasis [34]. It is reported that overexpressing SLC31A1 could increase copper uptake in breast cancer cells and xenograft models [35] and could also promote cuproptosis in kidney and lung cancer cell lines [11]. Furthermore, it was found that copper-induced cell death caused by the overexpression of SLC31A1 could not be prevented by ferroptosis, necroptosis, and apoptosis inhibitors, but could be partially rescued by copper chelators [11]. This indicated that SLC31A1 plays an important role in cuproptosis. At present, SLC31A1 might be a promising diagnostic/prognostic biomarker in breast cancer [36]; it has also been found to modulate chemotherapeutic sensitivity in other cancers [37,38]. LIAS (lipoic acid synthetase) has been found to involve the synthesis of mitochondria-associated metabolic enzymes, as well as energy metabolism and antioxidant responses [39]. Therefore, its mutations could result in the disorder of mitochondrial energy metabolism. It is reported that the cuproptosis-related biomarker LIAS plays a vital role in the progression of various cancers, and exploring its underlying mechanisms and biological functions may bring new hope to cancer treatment. Its high expression in patients with kidney renal clear-cell carcinoma, rectum adenocarcinoma, breast cancer, and ovarian cancer indicates a good prognosis, whereas in lung cancer patients it showed a poor prognosis. In addition, LIAS expression could predict the efficacy of cancer immunotherapy [40]. As a mitochondrial protein, DLD (dihydrolipoamide dehydrogenase) is an essential component for the multi-enzyme complexes that regulate energy metabolism. In addition, DLD can be used as a flavin protein oxidoreductase to bind to FAD by proton- and electron-catalyzed disulfide bonds [41]. Shin et al. [42] found that DLD is closely related to cystine deprivation-induced ferroptosis, and inhibiting DLD could reduce lipid peroxidation and ferrous iron accumulation, thus inhibiting ferroptosis suppression in head and neck cancer. MTF1 (metal regulatory transcription factor 1) is a conserved metal-binding transcription factor in eukaryotes, which is sensitive to both metal excess and deprivation, and could promote myogenesis in response to Cu [43]. It is reported that lung adenocarcinoma cells lacking MTF1 will be more sensitive to oxidative stress [44]. CDKN2A (cyclin-dependent kinase inhibitor 2A) is a cyclin-dependent kinase inhibitor gene that encodes the p16 gene involved in a series of cell cycle pathways, such as inhibiting cell proliferation, promoting tumor cell apoptosis, and increasing tumor cell chemotherapy sensitivity by causing cell cycle arrest in the G1 phase. Recent research found that CDKN2A can not only regulate ferroptosis but also participates in the process of cuproptosis [11]. Recent studies have shown that CDKN2A is associated with poor prognoses in various cancers. Chen et al. [45] found that CDKN2A may be a potential prognostic predictor and the target of immunotherapy in triple-negative breast cancer. Luo et al. [46] found that CDKN2A expression may be conducive to regulating tumor-associated macrophages and could predict prognosis and immune infiltration in hepatocellular carcinoma. GCSH (glycine cleavage system protein H) is an enzymatically inactive cofactor of the other three glycine cleavage system enzymes and was up-regulated in thyroid cancer, lung cancer, breast cancer tissues. It has been reported that GCSH is a vital factor in maintaining the cellular metabolic status and viability, indicating that it is a critical biomarker in breast cancer cells [47]. According to our risk signatures, the patients were classified into low- and high-risk groups based on the risk scores. Kaplan–Meier survival curves showed that the OS of low-risk patients was longer than that of the high-risk groups. The AUC of the ROC curve showed that the risk signature was accurate in predicting the prognosis of THCA patients. Univariate and multivariate Cox regression analyses suggested that the risk score was not only an independent risk factor for prognosis but could also predict the clinical characteristics of THCA.

The functional enrichment analyses demonstrated that immune-related signaling pathways were highly enriched in the low-risk groups. Therefore, we speculate that cuproptosis may regulate the immune status and immune function in THCA. Firstly, the ESTIMATE algorithm was performed to evaluate the tumor purity of each sample. The stromal and immune scores were significantly higher in low-risk groups, indicating that the high-risk has an accordingly higher tumor purity, which is generally related to the poor prognosis. The immune response plays an indelible role in tumorigenesis and has often been used as a target for tumor therapy. In addition, we clarified the infiltration of immune cells in THCA and found that most immune cells, such as activated B cells, CD4 T cells, and CD8 T cells, were highly expressed in low-risk patients. In our previous study, we constructed a prognostic signature of eight immune-related genes to predict survival and response to ICIs in PTC [19]. Gan et al. [48] developed and validated a three-immune-gene model to predict outcomes in PTC. Moreover, an early transcriptomic study identified and specified the expression variability of hybrid genes related to the immune response in PTC [49]. Cancer immunotherapy is becoming a potential therapeutic agent and has demonstrated great progress in the cancer treatment field [20]. Therefore, the response of common immune checkpoint inhibitors was explored and the expression levels of PD-1, PD-L1, PD-L2, CTLA4, TIGIT, TIM-3, and LAG-3 were significantly higher in low-risk patients. Therefore, we could speculate that low-risk patients might have a more beneficial response to the treatment of these checkpoint inhibitors. Some new evidence suggests that Cu and its transporter could influence target therapy [30]. Therefore, we predicted the sensitivity towards targeted therapies in both low- and high-risk groups. We found that patients in the low-risk group were more sensitive to sorafenib, which might help in choosing appropriate drugs. In contrast, sunitinib and pazopanib are more sensitive to patients in the high-risk group. However, we verified the six signature genes’ expression in 50 pairs of PTC tissues and their adjacent non-cancerous thyroid tissue by qRT-PCR and obtained almost the same results as TCGA. However, it is still necessary to further expand the sample size and verify the potential mechanism of these risk CRGs in thyroid cancer cell lines.

Currently, there has been no study of cuproptosis in the thyroid cancer field; thus, it was of great significance to carry out this study. Our study on CRG risk signatures lays a theoretical foundation for future mechanism research. However, our study still has some limitations to be illustrated. All analyses were performed in a TCGA cohort, and only internal verification was carried out. A larger external validation cohort should be used to verify the predictive performance of our risk signature in THCA. Furthermore, some basic experiments are still required to investigate the correlation between the risk signature and the immune function.

## 5. Conclusions

In conclusion, we constructed a cuproptosis-related prognostic signature of genes that exhibited predictive efficiency based on a comprehensive analysis of RNA sequencing data and clinical data of THCA from a TCGA database. The signature was significantly associated with tumor immunity. Our study presented a better understanding of the relationship between cuproptosis and immunotherapy response in THCA patients. Our cuproptosis-related signature can provide new possibilities for predicting survival and create new expectations for personalized targeted therapy in THCA patients.

## Figures and Tables

**Table 1:** The clinical characteristics in the training, the testing, and the entire sets.

Variables	Type	The Entire Set (*n* = 502)	The Training Set (*n* = 251)	The Testing Set (*n* = 251)	*p* Value
Age	<55	336 (66.93%)	170 (67.73%)	166 (66.14%)	0.7043
	≥55	166 (33.07%)	81 (32.27%)	85 (33.86%)	
Gender	Female	367 (73.11%)	181 (72.11%)	186 (74.10%)	0.6148
	Male	135 (26.89%)	70 (27.89%)	65 (25.90%)	
Stage	Stage I-II	335 (66.73%)	171 (68.13%)	164 (65.34%)	0.5073
	Stage III–IV	167 (33.27%)	80 (31.87%)	87 (34.66%)	
T	T1-2	307 (61.15%)	157 (62.55%)	150 (59.76%)	0.5202
	T3-4	193 (38.45%)	93 (37.05%)	100 (39.84%)	
	Unknown	2 (0.40%)	1 (0.40%)	1 (0.40%)	
M	M0	281 (55.98%)	137 (54.58%)	144 (57.37%)	0.6879
	M1	9 (1.79%)	5 (1.99%)	4 (1.59%)	
	Unknown	212 (42.23%)	109 (43.43%)	103 (41.04%)	
N	N0	228 (45.42%)	114 (45.42%)	114 (45.42%)	0.6028
	N1	225 (44.82%)	107 (42.63%)	118 (47.01%)	
	Unknown	49 (9.76%)	30 (11.95%)	19 (7.57%)	

## Data Availability

We downloaded all data utilized in this research from the GDC portal (https://portal.gdc.cancer.gov/, accessed on 18 May 2022).

## Supplementary Materials

The following supporting information can be downloaded at: https://www.mdpi.com/article/10.3390/jcm12052014/s1, Table S1. The sequence of primers; Figure S1. The relationships between risk signature and clinic-pathologic parameters (age (A), gender (B), T stage (C), N stage (D), M stage (E), and clinical stage (F)). (G) Heatmap showing the connections between clinicopathologic factors and high- and low-risk groups; Figure S2. Stratified analysis for the predictive efficacy of the cuproptosis-related gene signature among the THCA patients in TCGA cohort.
