# Supplementary figures and images for: Identification of a Novel Cuproptosis-Related Gene Signature and Integrative Analyses in Thyroid Cancer
Source: J Clin Med. 2023 Mar 3;12(5):2014. doi: 10.3390/jcm12052014 (PMC10004009; doi:10.3390/jcm12052014)

Figure S1

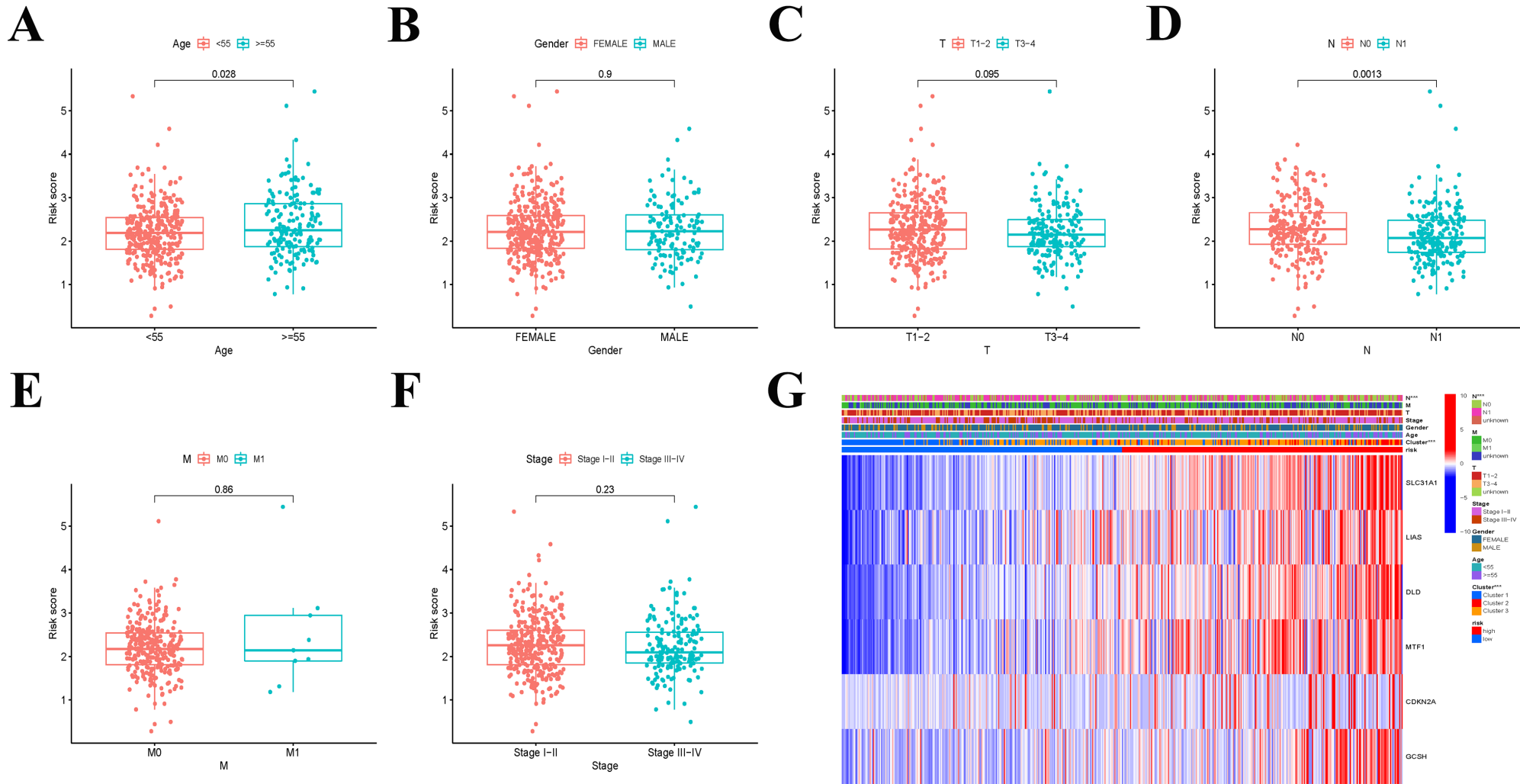

# Figure S2

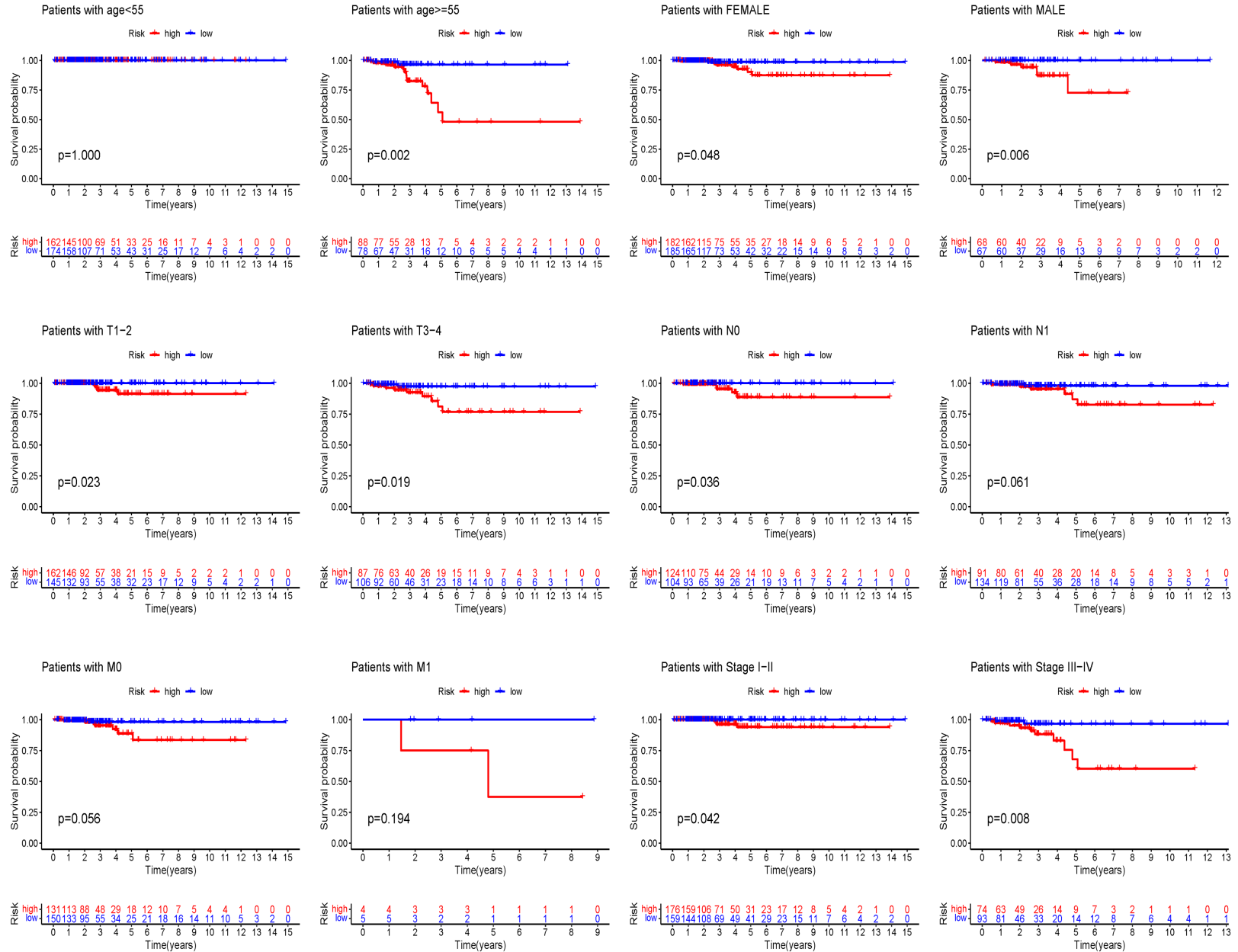

Supplement: Supplementary file 1 [file jcm-12-02014-s001.zip › jcm-2207735-supplementary.pdf]
